# The impact of traditional Chinese paper-cutting in digital protection for intangible cultural heritage under virtual reality technology

**DOI:** 10.1016/j.heliyon.2024.e38073

**Published:** 2024-09-18

**Authors:** Lulu Zhao, JaeWoong Kim

**Affiliations:** Graduate School of Advanced Imaging Science, Multimedia and Film, Chung-Ang University, Seoul, 100-744, South Korea

**Keywords:** Virtual reality technology, Intangible cultural heritage, Paper-cutting, Digital protection, Three-dimensional modeling, Questionnaires

## Abstract

Under the impact of the digital wave, traditional paper-cutting art faces unprecedented challenges in preservation and dissemination. Given the urgent need for Intangible Cultural Heritage (ICH) protection, this study explores the application of Virtual Reality (VR) technology in the preservation of paper-cutting art. VR efficiently digitizes and widely disseminates paper-cutting art by skillfully integrating modern technological elements. The study not only deeply analyzes how VR technology can safeguard and inherit traditional Chinese paper-cutting art but also focuses on the meticulous design and optimization of the paper-cutting VR system. The constructed VR system integrates cutting-edge technologies such as 3D modeling and modular design. It showcases outstanding performance in virtual environment rendering and user interaction, rigorously validated through precise evaluation methods. Experimental results demonstrate the paper-cutting VR system's response time of only 10 ms and a high frame rate of 90 frames per second, highlighting its exceptional performance. Furthermore, comprehensive research on the profound impact of the VR system on various stakeholders contributes a new perspective and profound insights into the digital preservation of ICH. In summary, this study successfully innovatively applies VR technology to the protection and dissemination of paper-cutting art, achieving digital preservation goals and injecting new vitality into its inheritance. This pioneering research path opens a hopeful new channel for the digital preservation of ICH and provides a valuable reference for the conservation of other traditional cultures. The study holds critical academic value and significant practical implications. Moreover, the extensive prospects of VR technology in the cultural heritage conservation field urgently require further exploration and investigation.

## 1 Introduction

In today's rapidly developing society, the preservation and transmission of traditional paper-cutting art face unprecedented challenges. These challenges are apparent in the gradual erosion of skills, shifts in traditional craft environments, and the profound influence of modernization on Intangible Cultural Heritage (ICH) [1,2]. As a testament to human ingenuity and creativity, safeguarding and passing down ICH are crucial for honoring cultural diversity, and for respecting and preserving history. However, as modernization advances, many forms of ICH face threats such as reduction, loss, and forgetfulness [3,4], heightening the urgency of protecting and transmitting traditional paper-cutting art. As a significant representative of China's ICH, traditional paper-cutting art possesses profound artistic value and historical significance [5,6]. Despite this, its transmission process encounters numerous challenges. The loss of skills, changes in the craftsmanship environment, and the inefficiency and difficulty in promoting traditional teaching methods severely impede the transmission and evolution of paper-cutting art. To tackle these challenges, innovative solutions are urgently required to comprehensively protect and effectively transmit paper-cutting art. In this regard, virtual reality (VR) technology, with its unique interactivity and immersion, furnishes new possibilities for the digital preservation and transmission of traditional paper-cutting art. VR technology can construct lifelike virtual environments where participants can engage firsthand in the paper-cutting process and appreciate its unique artistic allure [7]. This immersive learning experience not only enhances participants' comprehension and proficiency in ICH but also sparks their interest and awareness in its preservation and transmission. Traditional recording methods, such as textual descriptions and photographic images, can present the characteristics of paper-cutting art but cannot fully capture its three-dimensional (3D) and dynamic beauty. Physical preservation is constrained by space, time, and environment, making it difficult to maintain the intricate craftsmanship of paper-cutting for extended periods. However, VR technology can overcome these limitations by showcasing the beauty and cultural value of paper-cutting art in a new way. Based on VR technology, the 3D forms and dynamic effects of paper-cutting works can be recreated, allowing viewers to experience the charm of paper-cutting art in a virtual environment. Furthermore, traditional methods of teaching paper-cutting skills are often limited by time and space, making it difficult for a broader audience to experience and learn firsthand. VR technology can overcome these barriers by providing a new learning platform for learners. By constructing an interactive VR paper-cutting system, learners can freely explore and practice in a virtual environment, thus enhancing learning outcomes and interest. In summary, this study aims to confront the challenges associated with the transmission of traditional paper-cutting art by introducing VR technology as an innovative solution for its digital preservation and transmission. By leveraging VR technology, the goal is to achieve comprehensive protection and effective transmission of paper-cutting art, fostering its preservation and advancement in contemporary society, enhancing cultural diversity, and facilitating the harmonious integration of tradition with modernity.

Firstly, in contrast to traditional digital recording methods like photography and videography, VR technology offers a more immersive experience. Users can engage with static images of paper-cutting works and interact with them in a virtual environment, experiencing the process and enjoyment of paper-cutting firsthand. This immersive capability grants VR technology distinct advantages in the ICH preservation field. Secondly, unlike augmented reality (AR) technology, VR technology establishes a wholly virtual environment, eliminating the necessity for users to switch between the real world and virtual information. In transmitting paper-cutting art, VR technology can provide users with a pure, distraction-free learning environment, helping them focus better on learning and experiencing the art. Additionally, compared to artificial intelligence (AI) technology, the application of VR technology in ICH preservation emphasizes direct user participation and experience. While AI can optimize learning experiences through algorithms and data analysis, the immersive experience provided by VR is difficult to replicate with AI alone. VR enables users to personally experience the charm of traditional culture, thus stimulating their interest and enthusiasm for ICH preservation. Finally, compared to 3D printing technology, VR technology concentrates more on the transmission and experience of culture rather than the replication of physical entities. VR can simulate the authentic paper-cutting process, enabling users to learn and master paper-cutting techniques in a virtual environment without relying on physical tools and materials. These characteristics give VR technology broader application prospects in ICH preservation.

**Fig. 1:**
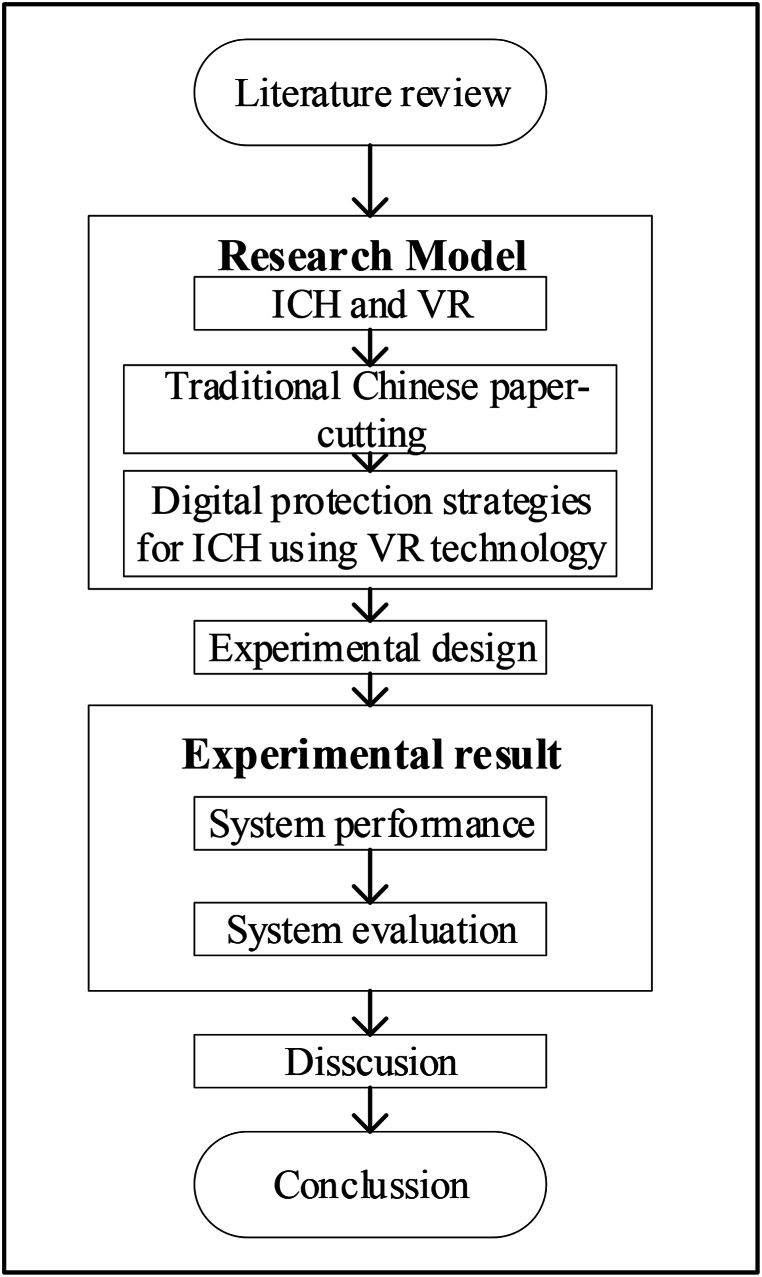
The research structure of this work.

Therefore, the core question of this study is how to effectively introduce VR technology to digitize and protect traditional Chinese paper-cutting art, thereby enhancing the transmission of this traditional craft. Specifically, this study delves into the design, optimization, and evaluation of a paper-cutting VR interactive simulation system, aiming to improve the ICH preservation levels. Through detailed case studies, the study evaluates the system's performance in virtual environment rendering, controller interaction, physical simulation, and paper folding modules. This study also investigates how these components impact different participant groups, including men, women, ICH preservation professionals, and the general public. The study's structure is well-defined. Initially, it explores the innovative applications of VR technology in the ICH field, highlighting traditional Chinese paper-cutting as a prime example. Subsequently, by designing and evaluating the paper-cutting VR interactive simulation system, it details how the virtual environment, interaction modules, and physical simulation jointly enhance the digitization and preservation of ICH. Lastly, the study focuses on the participation and feedback of different groups, providing a more comprehensive perspective on the research outcomes. This study makes specific contributions by proposing and executing an innovative approach to applying VR technology in ICH preservation, focusing notably on traditional Chinese paper-cutting art. Through meticulous design and comprehensive evaluation, this study opens new avenues for the digital preservation of ICH and offers valuable insights for future developments in this domain. Furthermore, examining the effects on diverse participant groups enriches the understanding of preservation efforts and offers useful insights for related research and practice. Fig. 1 presents this study's specific structure.

## 2 Literature review

At the international level, VR technology was widely applied in the digital preservation of ICH. Many scholars made remarkable contributions to the development of this field through in-depth research and exploration. First, Ahuja et al. (2023) successfully developed a VR-based traditional art experience system [8]. This system utilized high-fidelity 3D modeling and rendering technology to accurately reconstruct the details and scenes of traditional artworks, furnishing users with an immersive experience. Moreover, they integrated interactive controller modules, allowing users to interact with traditional artworks, thus deepening their experience of traditional culture. Additionally, Trček (2022) employed VR technology to recreate traditional music performances and dances [9]. The performances of traditional musicians and dancers were successfully reproduced through detailed audio and animation models. This innovative application not only enabled users to closely appreciate the splendid performances of traditional arts but also offered them a novel approach to learning and inheriting these cultural practices.

In China, scholars were also actively exploring the application of VR technology in ICH protection, following the international trend. Liu et al. (2022) utilized VR technology to reconstruct traditional opera performance scenes, providing audiences with an immersive experience of opera appreciation [10]. This novel approach allowed audiences to experience the charm of traditional opera firsthand and deepened their understanding and appreciation of traditional culture. Furthermore, Zhang et al. (2022) utilized VR technology to restore ancient architecture and cultural relics. Through precise 3D modeling and texture mapping techniques, they achieved a highly faithful reproduction of ancient cultural heritage [11]. Through virtual restoration, participants could explore and learn about ancient cultural heritage in a virtual environment, experiencing the richness and charm of historical culture firsthand. Li (2022) developed a virtual ICH inheritance system combining gesture recognition and body tracking technology [12]. This system enabled participants to directly experience the inheritance process of ICH by simulating traditional techniques and gestures, thereby imparting traditional skills to them. This technological innovation injected new vitality into the inheritance of ICH, making it easier for the younger generation to approach and understand the essence of ICH. Shoetan and Familoni (2024) studied the CHROMATA platform, which aimed to foster immersive ICH experiences by leveraging advanced extended-reality interaction technologies and AI [13]. This platform streamlined content retrieval from online resources and cultural institutions, offered AI-powered multimedia analysis services (including 3D pose estimation, folk dance recognition, and text analysis), and featured a user-friendly design interface for non-developers. By integrating images, videos, text, audio, and 3D models analysis with advanced 3D reconstruction technology, CHROMATA revitalized ICH by streamlining the creation of immersive experiences. The platform's development was supported and validated through practical cases of Greek cultural customs and dance virtual experiences.

In summary, while scholars worldwide have made notable strides in utilizing VR technology for safeguarding ICH, current research predominantly focuses on enhancing cultural transmission through immersive heritage experiences. However, these studies often overlook critical system challenges such as limited interactivity and realism. The uniqueness of this study lies in its in-depth analysis of the specific case of traditional Chinese paper-cutting art. This not only offers a concrete illustration of ICH preservation within this domain but also underscores the practical application value of VR technology. Compared to existing research, this study emphasizes the importance of VR technology in ICH protection and proposes targeted solutions to current problems. This not only offers a concrete illustration of ICH preservation within this domain but also underscores the practical application value of VR technology. For instance, this study tackles issues related to insufficient interactivity and realism by optimizing virtual environment rendering and enhancing interactive module performance. Additionally, it delves into virtual environment rendering, interactive modules, paper folding, and physical simulation, enhancing the practical utility of the ICH digital protection system through rigorous performance testing and optimization. These efforts not only improve system performance and user experience but also introduce novel methods and perspectives for ICH digital protection. Furthermore, this study highlights the active interest of domestic scholars in leveraging VR technology for ICH preservation, presenting fresh insights and practical approaches for safeguarding and transmitting domestic cultural heritage. Building upon international research achievements, this study contributes new perspectives and methodologies for employing VR technology in ICH digital protection through detailed case studies and performance analyses, aiming to advance the development of cultural transmission. In conclusion, through a comprehensive literature review and technical discourse, this study meticulously examines the shortcomings of current research and proposes targeted solutions and enhancement measures based on specific cases and performance evaluations. Consequently, it enriches research content quality and charts a new course and perspective for VR technology applications in the realm of ICH protection.

## 3 Research model

### 3.1 Research technology basis

The proposed technical overview mainly focuses on applying VR technology in the digital protection and inheritance of traditional Chinese paper-cutting art. Through in-depth analysis and careful design, the technology solves the inheritance and protection problems faced by traditional paper-cutting art in the process of modernization and opens up a new way for the protection of ICH.

(1) Technical overview and technical framework

At its core, the proposed technology focuses on constructing a comprehensive and highly realistic virtual environment on the VR platform. This entails integrating multiple modules seamlessly, including virtual environment rendering, controller interaction, physical simulation, and paper folding simulation. Together, these components provide users with an immersive exploration of the intricate art of paper-cutting. The virtual environment rendering module employs advanced graphics rendering techniques coupled with sophisticated lighting and material effects algorithms. This ensures that the virtual scenes and tools depict an exceptionally lifelike representation of a paper-cutting workshop. Users can closely observe each detail and appreciate the unique charm of paper-cutting art as if they were physically present. The controller interaction module incorporates advanced interaction algorithms, such as gesture recognition and voice recognition technology. This enables users to interact naturally with virtual elements, enhancing operational ease, user engagement, and immersion. Users can effortlessly manipulate virtual tools to create intricate paper-cutting works. In the physical simulation module, an advanced physics engine accurately simulates the real paper-cutting process, including texture, weight, and toughness. This simulation replicates physical phenomena such as bending, folding, and tearing, providing users with a realistic learning experience that enhances their understanding and interest in paper-cutting art. The paper folding module enables precise control and simulation of paper through complex algorithmic design. Users can perform detailed paper folding operations in the virtual environment, such as folding, curling, and flipping, enhancing operational accuracy and flexibility. This capability facilitates efficient learning and mastery of paper-cutting techniques, thereby improving the efficacy of transmitting paper-cutting art. In summary, the meticulously designed algorithmic structure of the proposed technology integrates VR with traditional paper-cutting art, delivering an unprecedented immersive experience. This innovation not only provides a new avenue for transmitting traditional paper-cutting art but also offers fresh perspectives for safeguarding ICH.

(2) Innovation point

The combination of VR technology and traditional paper-cutting art: This study applies VR technology to the digital protection and inheritance of traditional paper-cutting art for the first time. By constructing a highly realistic virtual environment, users can immerse themselves in the production process and artistic allure of paper-cutting art. This innovative integration offers a fresh approach to preserving paper-cutting art and introduces new paradigms for safeguarding ICH.

a. Advanced interactive technology and physical simulation technology: This study adopts advanced interactive technology and physical simulation technology, enabling users to more easily interact with the elements in the virtual environment, and feel the real paper-cutting process. This technology enhances the user's sense of participation and immersion, making users more deeply understand and learn the paper-cutting art.
b. Consideration of different types of participants: In the design and evaluation process, this study fully considers the needs and characteristics of participants, covering men, women, professionals from ICH protection units, and the public. This consideration makes the technology more widely applicable and popular, supporting the inheritance and popularization of paper-cutting art.

In conclusion, the proposed technology holds significant innovative and practical value in digitally preserving and transmitting traditional paper-cutting art. Through the promotion and application of this technology, this precious ICH can be better protected and inherited, and make greater contributions to the protection and inheritance of cultural diversity.

### 3.2 ICH and VR

This section introduces some theoretical foundations required for research, involving the relevant theories of ICH and VR technology, laying a theoretical foundation for subsequent research.

(1) ICH

ICH refers to intangible forms of cultural expression, such as oral traditions, performing arts, social practices, and traditional handicrafts created by human beings and passed down from generation to generation [[14], [15], [16]]. It represents spiritual and cultural wealth, comprising traditional knowledge, skills, customs, celebrations, festivals, and the material cultural artifacts and sites associated with them [[17], [18], [19]]. ICH embodies the unique identity, historical memory, values, and traditional wisdom of a community or a people. It is a vital manifestation of cultural diversity and human creativity [20,21]. Table 1 displays some common categories of ICH.

**Table 1:** Common categories of Ich.

Categories	Description
Oral tradition	Oral literature, folk tales, proverbs, and riddles
Performing arts	Traditional music, dance, theater, and folk performances
Social practices	Social customs, rituals, festivals, and religious ceremonies
Traditional handicrafts	Traditional craft skills, textiles, ceramics, wood carving, and gold and silverware
Traditional knowledge and skills	Knowledge of traditional medicine, farming techniques, building skills, and cooking skills

Table 1 categorizes the common ICH categories, including oral traditions, performing arts, social practices, traditional handicrafts, and traditional knowledge and skills. These categories represent the diversity and richness of ICH, underscoring the necessity for their protection and inheritance.

(2) ICH digitization

ICH digitization involves utilizing digital technology to collect, manage, store, and display ICH resources to protect, restore, and study these valuable items [22,23]. This process employs digital information technology to collect, store, display, and disseminate relevant information about ICH items, enabling their transformation and reproduction. This innovative approach enhances the protection of ICH [24,25]. Currently, various ICH items are digitized in different ways, as outlined in Table 2.

**Table 2:** Digital protection methods for different ICH items.

ICH items	Digital protection methods	Technical tools	Implementing agency
Traditional music	3D sound recording and archiving	High-definition recording equipment and sound editing software	Cultural heritage protection and musicological research institutions
Oral traditions and folk tales	Digitized archival records	Digital recording equipment and transcription software	Oral history research institutes, libraries, and museums
Traditional dance	3D motion capture and modeling	Motion capture equipment and 3D modeling software	Dance research institutes and digital art companies
Traditional craftsmanship	VR interactive experience	VR devices and interactive software	Handicraft heritage center and college of culture and art
Ancient opera	Digital performance and communication	Camera equipment and video editing software	Opera research institutions and cultural media companies
Traditional medicinal diet	Online platforms and apps	Web platforms and mobile app development tools	Chinese medicine research institutes and health management companies

Table 2 denotes that the existing ICH projects primarily encompass oral traditions, folk tales, ancient opera, traditional music, dance, craftsmanship, and medicinal diet. These projects employ diverse digital preservation methods such as sound recording and archiving, digitizing archival records, 3D motion capture and modeling, VR interactive experiences, digital performance and communication, and online platforms and applications. Implemented by a range of institutions including cultural heritage preservation organizations, oral history research centers, dance research institutions, handicraft heritage centers, opera research institutions, Chinese medicine research institutions, and others, these efforts effectively safeguard and transmit ICH.

(3) VR technology

VR creates a simulated environment via computer technology, allowing users to interact and experience immersion [[26], [27], [28], [29]].

**Fig. 2:**
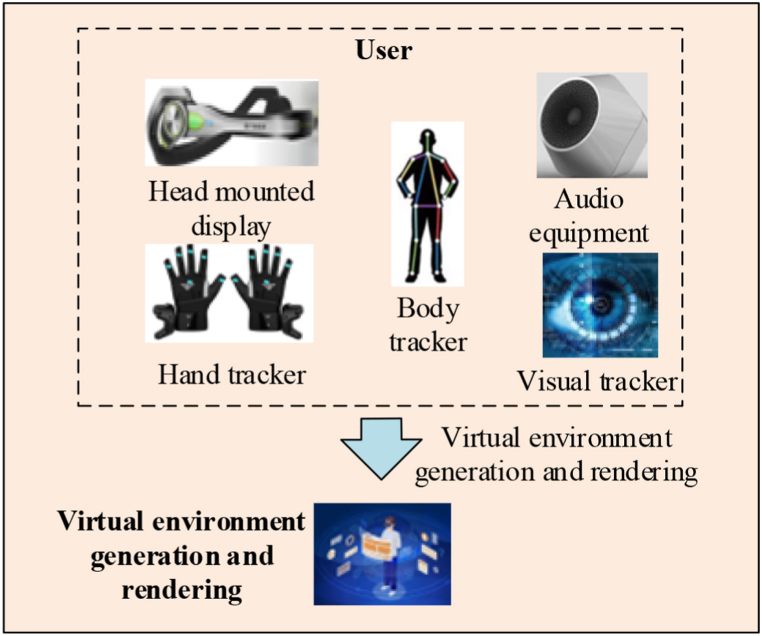
Schematic diagram of VR technology.

VR technology typically encompasses vital components, such as head-mounted displays, tracking systems, and input devices [30,31]. The relationship between the fundamental components of VR technology and user experience is depicted in Fig. 2.

**Fig. 3:**
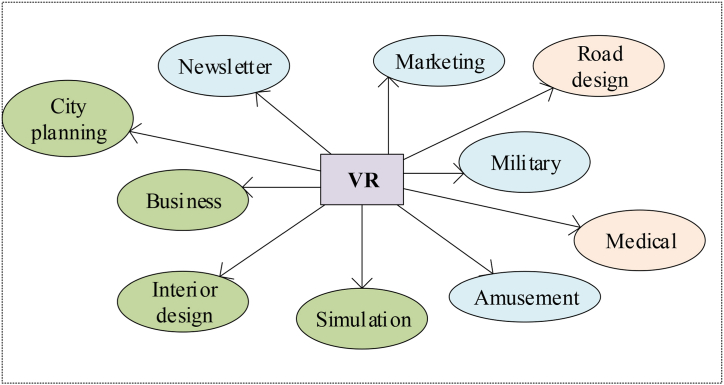
VR technology application fields.

Fig. 2 depicts the user's entry into a virtual environment via a headset. The tracking system monitors both head and body movements, and an input device enables interaction within the virtual environment. Specialized generation and rendering techniques present these virtual environments to users. Fig. 3 illustrates the widespread adoption of VR technology since its inception.

(4) Challenges faced by VR technology in the ICH digitization

Currently, VR technology encounters several challenges in ICH digitization. Firstly, its widespread adoption is impeded by high costs, complex equipment, and technical requirements, which limit its application on a larger scale [32,33]. Secondly, creating and maintaining content of high quality is a significant challenge. This involves translating the unique cultural value of ICH into content suitable for VR environments, requiring specialized expertise and skills [34]. Additionally, fostering user acceptance and engagement presents another obstacle. There is a need to enhance user acceptance of VR technology and encourage active participation in the digital preservation of ICH.

### 3.3 The history, skills, and protection status of traditional Chinese paper-cutting

This section scrutinizes the current state of protection for traditional Chinese paper-cutting culture, furnishing valuable insights into the potential integration of virtual technology with paper-cutting culture in the future.

(1) The origin and development of traditional Chinese paper-cutting

Traditional Chinese paper-cutting constitutes a venerable form of folk art that has endured over time. It is an artistic creation of fashioning paper into various shapes with scissors [35,36]. Table 3 delineates the inception and evolution of traditional Chinese paper-cutting.

**Table 3:** The origin and development of traditional Chinese paper-cutting.

Stages	Description	Features
Origin stage	A paper-cutting activity before the Han Dynasty for decoration and sacrificial ceremonies.	Simple paper-cutting and basic patterns and shapes.
Development stage	It develops into an independent art form with a wealth of subject matter and techniques.	Regional styles, such as window flowers and carved paper. It focuses on symmetry and proportion.
Application stage	It is widely used in festivals, weddings, and other occasions.	It expresses the important elements of blessing and good luck.
Contemporary stage	It is protected, passed on, and developed commercially.	It combines modern elements and artistic expression.

Table 3 exhibits that traditional Chinese paper-cutting originated before the Han Dynasty and was initially used for simple decoration and sacrificial ceremonies with simple basic patterns. Gradually, it evolved into a distinct art form with rich themes and techniques, giving rise to distinct regional styles. It is extensively applied in festivals, weddings, and other occasions, symbolizing blessings and good fortune. In contemporary society, traditional paper-cutting has been protected and inherited, developed commercially, combined with modern elements and artistic expression, and continues to prosper.

(2) Current situation and problems of traditional paper-cutting protection

Presently, the inheritance of traditional paper-cutting techniques faces the risk of interruption owing to societal transformations and shifts in modern lifestyles. Many seasoned paper-cutting artists confront the predicament of an absence of successors [37,38]. The traditional paper-cutting market has limited demand and lacks good commercial operation and market promotion, resulting in livelihood challenges for practitioners. Moreover, obstacles arise in the supply chain for paper-cutting materials and tools, with traditional handmade methods demanding considerable time and effort [[39], [40], [41], [42]].

### 3.4 Digital protection strategies for ICH using VR technology

Drawing upon the aforementioned theoretical framework of VR technology and the preservation status of Chinese paper-cutting culture, this section proposes a digital preservation strategy for paper-cutting employing VR technology.

(1) Design and implementation of digital protection strategy for paper-cutting based on VR technology
1) 3D modeling and virtual restoration of paper-cutting production process

To effectuate the amalgamation of VR technology with paper-cutting art, the initial step entails leveraging 3D modeling and VR technology to simulate and reconstruct the paper-cutting production process. Accordingly, Table 4 shows the VR reconstruction scheme for the paper-cutting production process, aligning with the characteristics of VR technology and its implementation process.

**Table 4:** Vr restoration scheme for the paper-cutting production process.

Steps	Name	Scheme content
Step 1	Data collection and conceptual design	Photos and videos of Chinese window flower paper-cutting works from different regions and styles are collected.
		It is determined to use traditional Chinese window flower paper-cutting as a case for virtual restoration.
		Paper-cutting patterns and shapes are designed, to identify key steps in the production process.
Step 2	3D modeling	A virtual environment using 3D modeling software is created, and a window flower paper-cutting model is imported.
		The drawing tools and curve tools are used to draw the contour lines and cutting paths of the window flower paper-cutting in the software.
Step 3	Materials and textures	The appropriate paper material is selected, and the texture is added to the virtual window grille decoupage model, simulating the texture and pattern of the real window grille decoupage.
Step 4	Animation and simulation	The cutting action of the scissors is simulated, and the virtual scissors are moved along the cutting path to cut the paper into a window pattern.
		Through the simulation effect, the virtual window flower paper-cutting model presents the dynamic effect of paper-cutting.
Step 5	Lighting and rendering	The appropriate lighting effects and rendering parameters for the virtual window flower paper-cutting model are set for realistic lighting and material reflections.
Step 6	Interactive experiences and user interactions	Virtual window flower paper-cutting models are imported into VR platforms or applications.

**Fig. 4:**
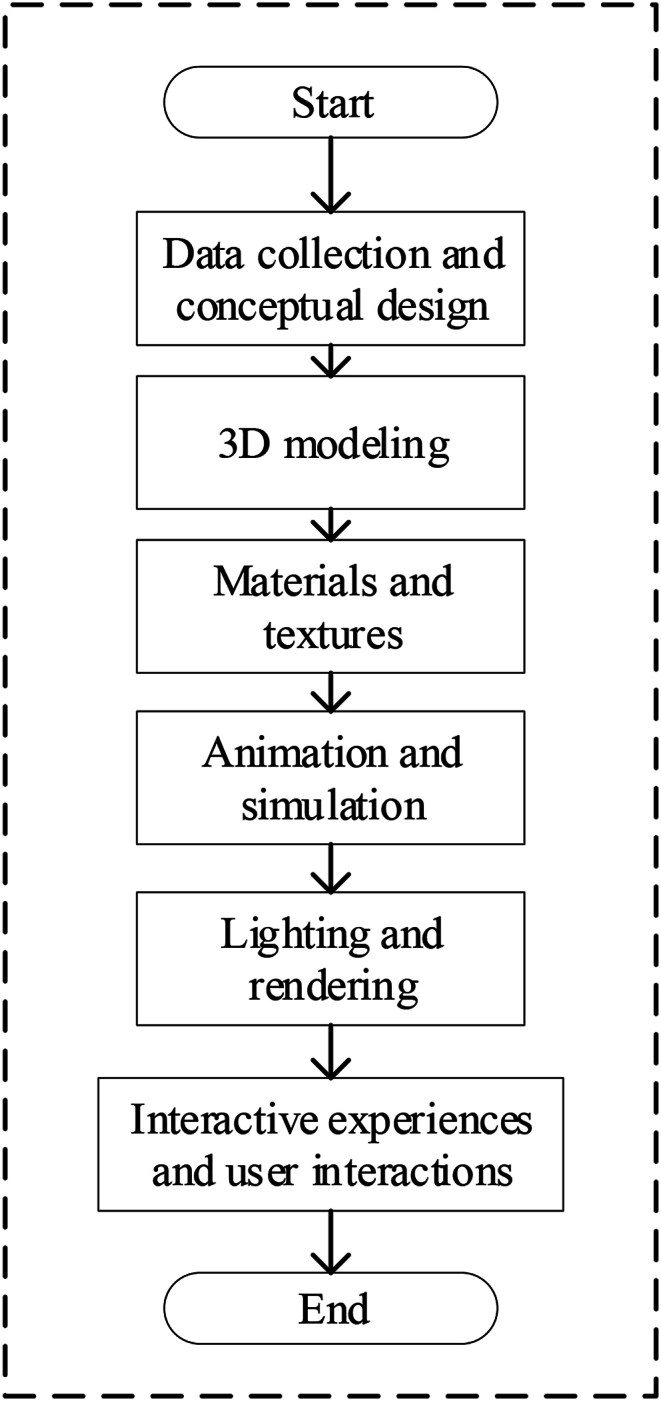
The VR restoration scheme of the paper-cutting production process.

**Fig. 5:**
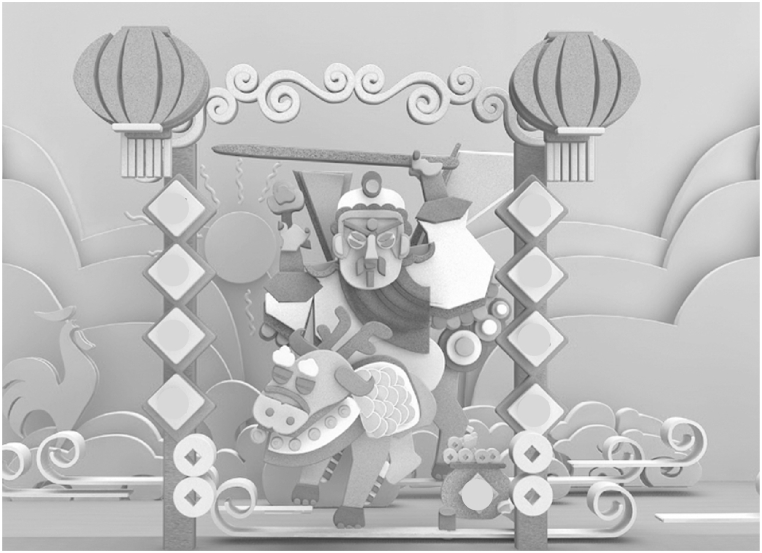
Design of paper-cutting based on VR.

**Fig. 6:**
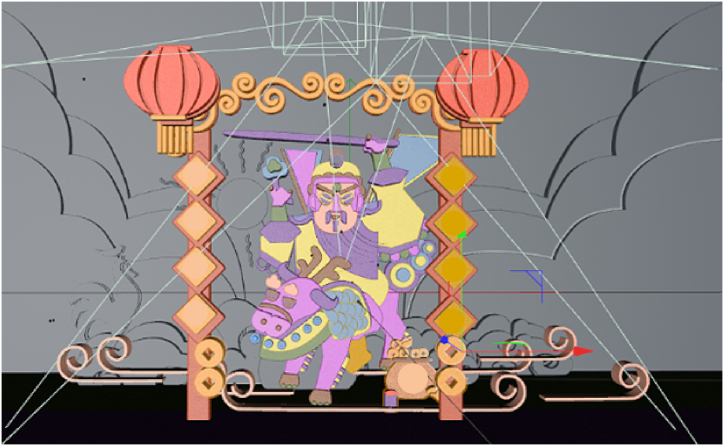
Results of paper-cutting based on VR.

The flow of the specific scheme and the process of paper-cutting using VR are depicted in Fig. 4, Fig. 5, Fig. 6.

2) Design of paper-cutting simulation module based on VR technology

**Fig. 7:**
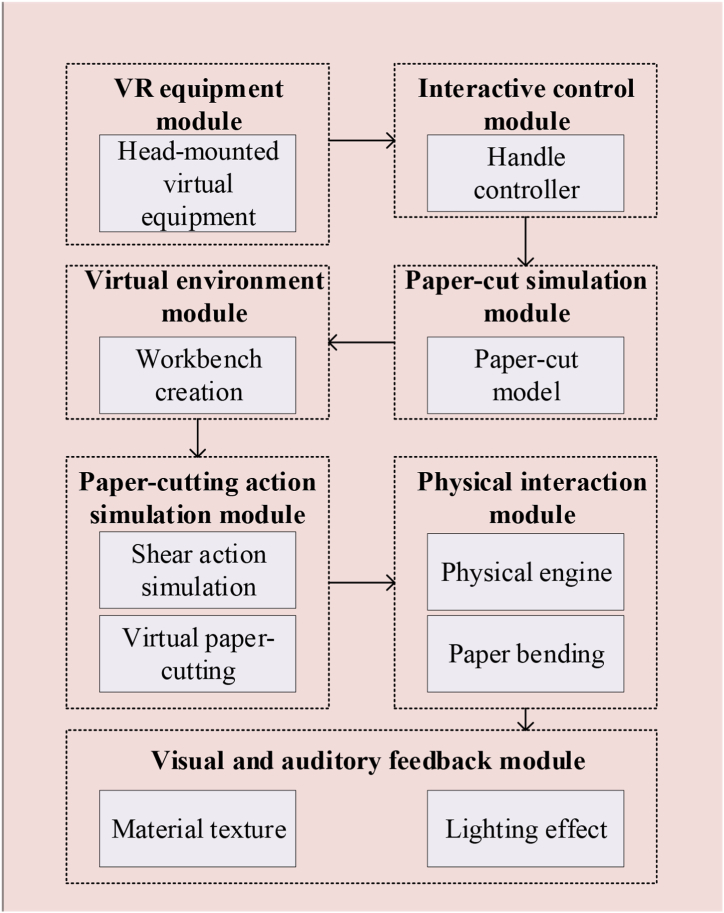
Schematic diagram of the design of the paper-cutting simulation module based on VR technology.

According to the above scheme, the schematic diagram of the VR technology-based paper-cutting simulation module is suggested in Fig. 7.

Fig. 7 denotes that in the paper-cutting simulation module based on VR technology, users enter the paper-cutting production workbench in the virtual environment by wearing a VR headset and operating handle and simulating the real paper-cutting process in VR [43].

3) VR module implementation of the paper-cutting process

First, to realize the designed traditional paper-cutting VR module, the technical framework and hardware and software environment parameters used here are demonstrated in Table 5.

**Table 5:** The implementation environment of the traditional paper-cutting Vr module.

Environment type	Parameter name	Configuration
Hardware environment	VR devices	Windows Mixed Reality Helmet
	Computer hardware	Intel Core i7 processor; NVIDIA GeForce RTX 30 graphics card; 16 GB memory
	VR interactive controller	Oculus Touch control
Software environment	VR development tools and engines	Unity
	3D modeling software	Blender
	Physical engine	PhysX

Table 5 suggests the technical framework, hardware, and software environment parameters required for the traditional paper-cutting VR module. These parameters are crucial for ensuring the fluidity, realism, and interactivity of the VR experience. Firstly, for the hardware environment, a Windows Mixed Reality headset serves as the VR device. This headset typically provides a wide field of view and high comfort, delivering an immersive experience for users. Paired with an Intel Core i7 processor and an NVIDIA GeForce RTX 30 series graphics card, this high configuration ensures the system can handle complex graphic rendering and physical simulations, guaranteeing a smooth and high-quality VR experience. The 16 GB memory offers sufficient space for multitasking and large projects. In addition, the Oculus Touch controllers are employed as VR interaction devices, offering intuitive gesture recognition and interaction methods that enhance user immersion. In terms of the software environment, Unity is chosen as the VR development tool and engine. Known for its powerful cross-platform development capabilities and extensive resource library, Unity greatly facilitates the development of VR applications. Blender, an open-source 3D modeling software, is used for its robust functionality and support for exporting in various formats, ensuring seamless integration with other software. The PhysX physics engine can simulate real-world physical effects such as gravity and collisions, furnishing users with a more realistic paper-cutting experience. In conclusion, the technical framework, hardware, and software environment parameters listed in Table 5 are optimized and selected to cater to the characteristics of the traditional paper-cutting VR module. These configurations provide users with a smooth, realistic, and highly interactive VR experience. They meet the current demands of VR technology development and offer robust support for future applications in preserving paper-cutting art and other cultural heritage fields [44].

**Fig. 8:**
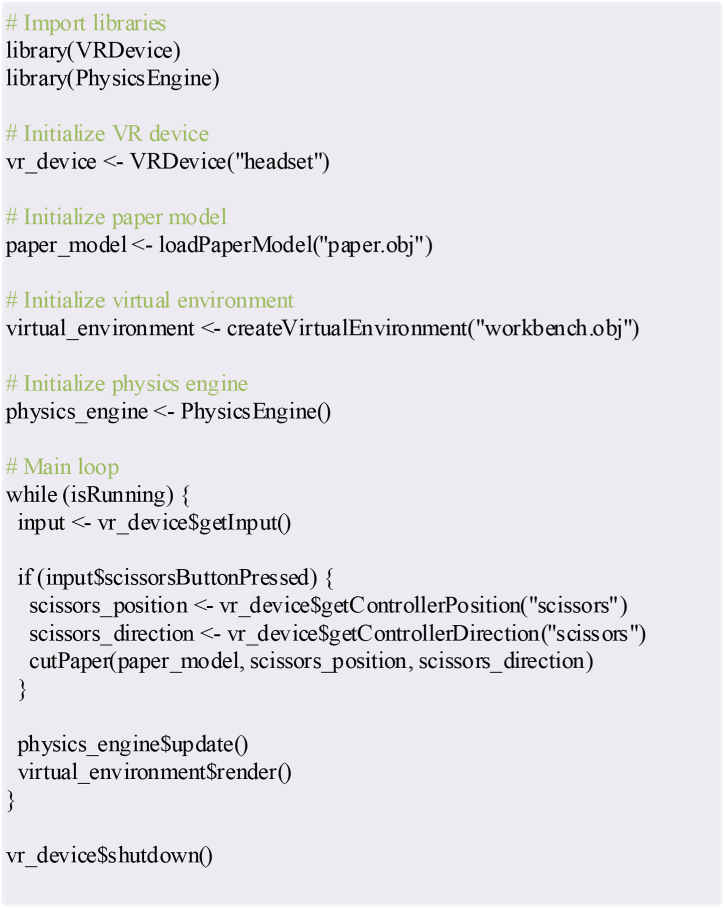
Pseudocode of paper-cutting simulation module based on VR technology.

In addition, the pseudocode design is used in the R language to realize the VR technology-based paper-cutting production simulation module. Fig. 8 presents the design results.

In Fig. 8, R language code facilitates physical simulations and interactive applications within a VR environment. The code is integrated into the VR device's library and the physical engine, initializing the VR device, virtual paper model, and virtual environment, subsequently entering the main loop. The code continually acquires input data from the VR device in the main loop. Upon detecting the activation of the scissors button, the code simulates the interaction between the scissors and the virtual paper, updates the physics engine, and renders the virtual environment. Finally, the VR device is deactivated. This code enhances users' interaction with virtual objects in a VR environment.

(2) Experimental design

Five expert evaluators engage with the paper-cutting VR interactive simulation system and give professional scores to the individual modules of the system.

### 3.5 Model framework and system

1. Framework model of the proposed system

The framework model of a paper-cutting digital protection system based on VR technology proposed in this study covers several key parts:

(1) Data collection and concept design unit: Photos and videos of Chinese window paper-cutting artworks from different regions and styles are extensively gathered. Traditional Chinese window paper-cutting is explicitly selected as a typical case for virtual restoration. Patterns and shapes of paper-cutting are meticulously designed, while core steps in the production process are accurately delineated.
(2) 3D modeling and virtual restoration module: 3D modeling software is utilized to construct virtual environments and smoothly import window paper-cutting models. Drawing and curve tools are employed to carefully outline the contours and cutting paths of window paper-cutting. Suitable paper materials are cautiously selected, and realistic textures are added to the virtual window paper-cutting models to faithfully replicate their texture characteristics and pattern styles. By vividly simulating scissor-cutting actions and moving virtual scissors along carefully planned cutting paths, exquisite window patterns are successfully created, dynamically showcasing their effects. Careful adjustments of lighting effects and rendering parameters are made to create realistic lighting atmospheres and material reflection effects.
(3) Paper-cutting simulation unit: Users wear VR headsets and operate controllers to enter the virtual environment's paper-cutting workstation, realistically simulating the process of paper-cutting creation.
(4) Technical implementation module: Windows Mixed Reality headsets are selected as the VR equipment, working in conjunction with an Intel Core i7 processor, NVIDIA GeForce RTX 30 series graphics card, 16 GB of RAM, and Oculus Touch controllers. At the software level, Unity is employed as the VR development tool and engine, utilizing Blender for 3D modeling and employing PhysX as the physics engine. Additionally, pseudo-code in R language is designed to enhance the simulation module for paper-cutting production, significantly improving user interaction with virtual objects in the VR environment.
2. Description of the integrated system description

The above modules work closely together to construct a comprehensive VR-based digital preservation integrated system for paper-cutting. In the data collection and concept design unit, comprehensive and in-depth data collection provides a solid foundation and clear direction for subsequent modeling and restoration work. Leveraging advanced techniques, the 3D modeling and virtual restoration module vividly reproduces the production process of paper-cutting, presenting this art form in a dynamic digital format. The paper-cutting simulation unit creates an excellent opportunity for users to experience paper-cutting production firsthand, greatly enhancing deep interaction with paper-cutting culture. The technical implementation module ensures smooth operation, high realism, and outstanding interactive performance across the entire system. For instance, in the 3D modeling and virtual restoration module, meticulous modeling and texture processing techniques authentically showcase the intricate lines and distinctive patterns of traditional paper-cutting, immersing users in realistic creative scenarios. In the paper-cutting simulation unit, users can freely engage in paper-cutting creation in a virtual environment, experiencing firsthand the charm and endless joy of paper-cutting artistry.

## 4 Experimental design and performance evaluation

### 4.1 Datasets collection

The preceding sections successfully apply advanced VR technology to simulate traditional paper-cutting crafts within ICH, creating a vivid, interactive paper-cutting VR simulation system. This study meticulously organizes and conducts a series of carefully designed experiments to further validate the system's effectiveness in practical applications.

In constructing the experimental framework, this study thoroughly considers the diversity and comprehensiveness of the dataset to ensure the thoroughness and objectivity of experimental results. Here is a more detailed explanation of the experimental dataset:

(1) Professional data from ICH protection institutions: This study specifically invited 50 experts and professionals from renowned and authoritative ICH protection institutions in China to participate in the testing. These individuals have an average of over 15 years of profound theoretical knowledge and extensive practical experience in ICH protection. They have deep insights into the historical heritage, technical evolution, and cultural significance of paper-cutting art, driven by a profound emotional connection. Their expert opinions and high-quality data cover various details of paper-cutting techniques, such as precision in knife skills and pattern design, playing an irreplaceable role in accurately assessing the system's performance and accuracy in professional domains.
(2) General public data: To comprehensively understand the system's acceptance and actual effectiveness among the public, this study recruited 200 ordinary individuals from different age groups, professions, and cultural backgrounds as experimental subjects. Participants' ages ranged from 18 to 60 years, with the 20–40 age group comprising the majority at 60 %. Professions included teachers (25 %), engineers (30 %), students (25 %), doctors (10 %), and workers (10 %), reflecting diverse cultural backgrounds including Han, Mongolian, Tibetan, and other ethnicities. Data collection involved distributing 200 meticulously designed questionnaires, conducting 100 in-depth user interviews, and performing 50 experimental operations. This resulted in comprehensive and rich data crucial for evaluating the system's usability, interactivity, and appeal. Participants' feedback authentically reflects the system's practical application among a broader audience.
(3) Third-party evaluation data from expert scholars: This study invited 10 experts and scholars from universities, research institutions, and related fields to serve as third-party evaluators in the experiments. They have an average of over 10 years of rich experience and profound expertise in heritage protection and VR technology applications. Moreover, they could comprehensively and deeply evaluate the system from multiple dimensions, including technological innovation, user experience comfort, and educational significance. Their evaluations provided forward-thinking and constructive suggestions and profound insights, aiding this study in a clear and deep understanding of the system's strengths and weaknesses. Hence, important and targeted references can be offered for subsequent improvements and optimizations.

To sum up, this study successfully constructs a comprehensive, objective, and sizable experimental dataset by carefully collecting diverse data from professionals, the general public, and experts across various fields. The total dataset comprises 260 samples, including 50 professional data samples, 200 public data samples, and 10 expert scholar evaluation samples. Through appropriate division into training and testing sets, this study provides robust support for evaluating the practical application effects of the paper-cutting VR interactive simulation system. The specific dataset segmentation results are indicated in Table 6.

**Table 6:** The specific feature distribution of the dataset.

Dataset type	Data source	Data feature	Data size (sample size)	Training set size	Test set size
Professional data from protection institutions	Renowned ICH protection institutions	Professional, authoritative, and in-depth knowledge	50	40	10
Public group data	Different ages, professions, and cultural backgrounds	Broadness, diversity, and practical application feedback	200	160	40
Expert and scholar evaluation	Experts and scholars in related fields	Professionalism, independence, and in-depth analysis	10	Not applicable	10 (all for evaluation)

### 4.2 Experimental environment

This section introduces the experimental environment, covering software tools, computational environments, and data analysis tools.

**Fig. 9:**
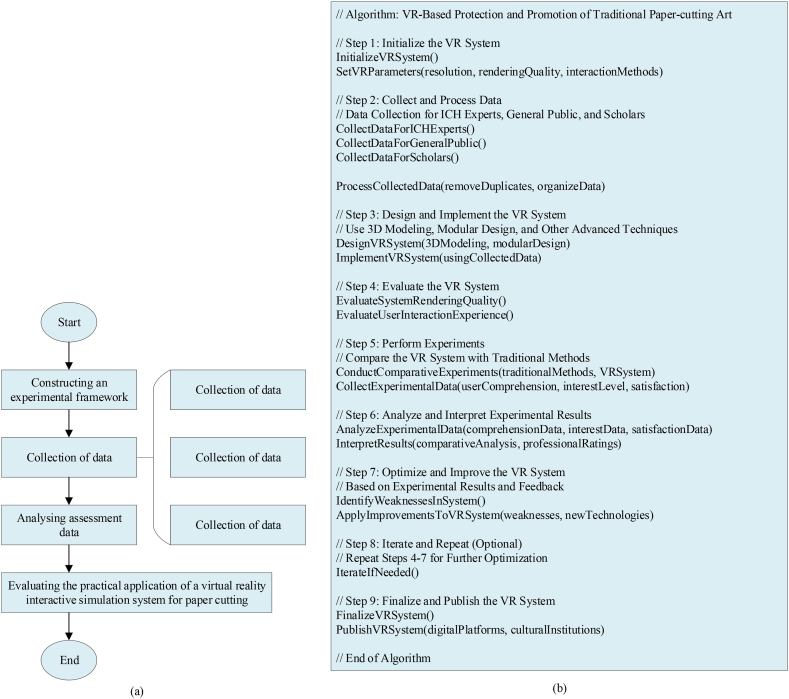
Flow chart and algorithm steps (a: flow chart; b: algorithm step).

Table 6 details the hardware and software environment utilized in the implementation of the paper-cutting VR interactive simulation system. To facilitate experiments for assessing the system's effectiveness, each experiment site is equipped with a dedicated computer capable of running VR applications and collecting experimental data. Meanwhile, a video camera or recording device is utilized to document the interactive experience and reactions of the participants. In addition, data analysis is conducted using the Statistical Package for the Social Sciences (SPSS) software. The flowchart and algorithmic steps are suggested in Fig. 9a and b.

Fig. 9 illustrates the systematic and rigorous approach required for scientific research and system development outlined in this study. Firstly, establishing the experimental framework is foundational. This step involves clearly defining the experiment's objectives, identifying key performance indicators for evaluation, and planning methods and sources for data collection. For instance, in the context of the paper-cutting VR interactive simulation system, it is crucial to specify metrics such as immersion, interactivity, and user satisfaction for assessment. At the same time, it is planned to collect data from professionals of ICH institutions, the general public, and relevant experts and scholars. Secondly, the process moves to the data collection stage. This stage includes inviting professionals from ICH institutions to provide evaluations and recommendations from a professional perspective. It also involves recruiting participants from diverse age groups, professions, and cultural backgrounds to collect their user experiences and feedback through surveys, practical operations, and interviews. Additionally, experts and scholars are invited to conduct third-party evaluations, analyzing the system from academic and technical perspectives. Finally, there is the data analysis and evaluation stage. Initially, all collected data undergo organization and preprocessing to remove invalid or anomalous data. Subsequently, appropriate data analysis methods such as statistical analysis, machine learning algorithms, etc., are applied to delve deeply into the data. For example, calculating means, variances, etc., are used to assess the system's performance stability; correlation analysis is employed to explore relationships between different factors; clustering analysis is utilized to classify user groups, understanding different user needs and behavioral patterns. Through these analyses and evaluations, an accurate assessment of the system's effectiveness in practical applications is achieved. The entire process is meticulously organized, showcasing standards in scientific research and system development, thus offering robust technical support and methodological guidance for the digital preservation and dissemination of paper-cutting art.

### 4.3 Parameters setting

This section outlines the key parameters of the experiment, focusing primarily on the scoring criteria essential for the study.

The evaluation indicators of this experiment are divided into two parts. The first part evaluates the designed system's performance, encompassing the response time, Frame per Second (FPS), and throughput.

In the second part of the experiment, 44 non-professional evaluators are divided into two groups, 22 in the control group and 22 in the experimental group. The control group undergoes the traditional paper-cutting teaching method, while the experimental group adopts the paper-cutting VR interactive simulation system. The experience time is 15 min. After the experiment, the effectiveness of the different paper-cutting culture experiences is evaluated through questionnaires. The questionnaire contains various indicators, scored on a scale of one to five, with five indicating the highest score and one the lowest. The final score is calculated based on the average rating from the participants. Additionally, the system's performance is evaluated post-test by five additional professional evaluators using a similar questionnaire to assess its effectiveness. Table 7 shows the design results of the parameters in this study.

**Table 7:** The design results of the parameters.

Parameter	Design details
System performance testing indicators	Response time, FPS, and throughput
Experimental grouping	22 people in the control group (traditional paper-cutting teaching method), 22 people in the experimental group (the paper-cutting VR interactive simulation system)
Experience duration	15 min
Scale of questionnaire ratings for non-professional evaluators	1-5 points (1 point is the lowest, 5 points are the highest)
Number of professional evaluators	5 people
The evaluation methods of professional evaluators	Questionnaire

### 4.4 Performance evaluation

This section analyzes and discusses the results of the experiment.

(1) System performance test effect

After the test, Table 8, Table underscore the performance test results of each module of the proposed system.

**Table 8:** Performance test results of each module of the paper-cutting Vr interactive simulation system.

Module name	Test indicators	Test result
Virtual environment rendering	Response time (ms)	10
	FPS	90
	Throughput (times/min)	120
User interaction	Response time (ms)	8
	Throughput (times/min)	60
Physical simulation	Response time (ms)	12
Paper folding	Response time (ms)	15

**Table:** 9Experimental time.

Experimental or analytical work	Estimated time spent (in weeks)	Actual time spent (in weeks)
Comparative experiments and collection and organization of results	4	4.5
Invite professionals to rate and summarize the results	2	2.1
Questionnaire survey and result analysis on different protection methods	3	3.2

Table 8, Table denote that after thoroughly examining the performance test results of each module of the paper-cutting VR interactive simulation system, it is evident that the entire system demonstrates exceptional efficiency. First, the virtual environment rendering module boasts a response time of just 10 ms, indicating that the system can swiftly respond to user commands, ensuring real-time interaction and fluidity. Additionally, with a frame rate of up to 90 fps, the system provides users with an almost lifelike visual experience, allowing the intricate details and textures of the paper-cutting art to be vividly displayed. In the user interaction module, the response time is an impressive 8 ms, underscoring the system's exceptional efficiency. This means that users hardly notice any delay when performing various operations, such as selecting tools or adjusting the viewpoint, significantly enhancing the sense of immersion. Moreover, achieving a throughput of 60 interactions per minute demonstrates the system's capability to swiftly process user inputs, ensuring seamless interactions. The physical simulation and paper-folding modules also exhibit commendable performance. With response times of 12 and 15 ms in specific modules, the system delivers real-time and precise simulation, enabling users to authentically experience the processes of paper-cutting and folding. The successful integration of these modules not only enhances the system's usability and enjoyment but also provides robust technical underpinning for the preservation and promotion of paper-cutting art. Overall, each module's performance test results in the paper-cutting VR interactive simulation system have reached high levels. This accomplishment is credited to the application of advanced technologies and meticulous system design. Through this system, users can deeply immerse themselves in the allure of paper-cutting art, gaining profound insights into its cultural significance and craftsmanship. Additionally, the system introduces innovative approaches for the preservation and transmission of paper-cutting art, highlighting its broad potential applications and significant societal impact. Additionally, there are slight discrepancies between the estimated and actual time spent on experimental or analytical work. Overall, the actual execution times are closely aligned with the estimates. For instance, while data collection and organization are estimated at 4 weeks, it takes 4.5 weeks in reality, likely due to unforeseen data issues during result compilation or the need for additional validation. The estimated time for professional evaluation and result compilation is 2 weeks, with actual completion in 2.1 weeks, demonstrating efficient execution of evaluation and compilation tasks. Finally, the estimated 3 weeks for surveying different preservation methods and analyzing results is exceeded slightly, taking 3.2 weeks. It indicates effective progress despite the time-intensive nature of surveying and data analysis.

(2) System evaluation effect
1) Analysis of comparative experimental results

**Fig. 10:**
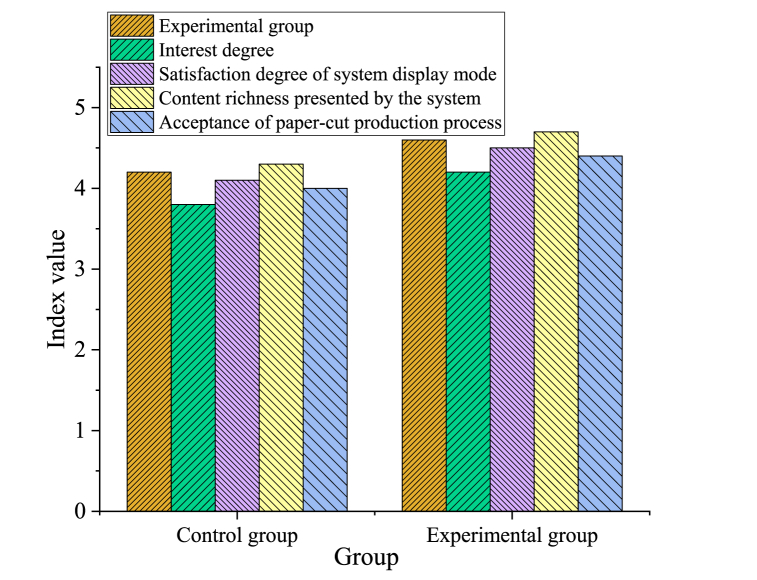
Comparison results of indicator evaluation in different groups.

After comparative experiments, the scoring results of various indicators of different groups of experimenters are plotted in Fig. 10.

Fig. 10 demonstrates that the average scores of the experimental group surpass those of the control group, affirming the designed paper-cutting VR interactive simulation system's superiority in facilitating paper-cutting culture compared to traditional paper-cutting teaching methods. Specifically, regarding the comprehension of paper-cutting culture, the average score of the experimental group reaches 4.6 points, while the control group scores only 4.2 points. This signifies that through the paper-cutting VR interactive simulation system, members of the experimental group have a deeper understanding and appreciation of paper-cutting culture, enabling them to better grasp the essence of paper-cutting art. Similarly, in terms of interest, the average score of the experimental group is also higher than that of the control group, reaching 4.2 points. This suggests that the designed system effectively fosters interest in paper-cutting culture among the experimental group, encouraging them more actively involved in paper-cutting activities. Considering satisfaction with the system presentation, the experimental group's average score exceeds that of the control group, at 4.5 points. This underscores the designed system's alignment with users' expectations and needs regarding interface design and interaction methods, providing them with a more comfortable and convenient experience. Additionally, regarding the richness of system content, the average score of the experimental group is notably higher, at 4.7 points, far exceeding the control group's 4.3 points. This reflects that the designed system can display more diverse and richer paper-cutting content, allowing users to explore various paper-cutting forms and techniques. Finally, considering the acceptance of the paper-cutting production process, the experimental group has an average score of 4.4, surpassing that of the control group. This indicates that the paper-cutting production process simulated by the designed system closely aligns with users' cognitive and operational habits, facilitating their acceptance and mastery of paper-cutting skills. In summary, these detailed data results fully demonstrate the designed system's advantages in promoting paper-cutting culture, demonstrating higher teaching effectiveness and user satisfaction compared to traditional paper-cutting teaching methods.

2) Professional scoring results

The system is scored by five professionals, and the subjective scoring results of the system are obtained.

**Fig. 11:**
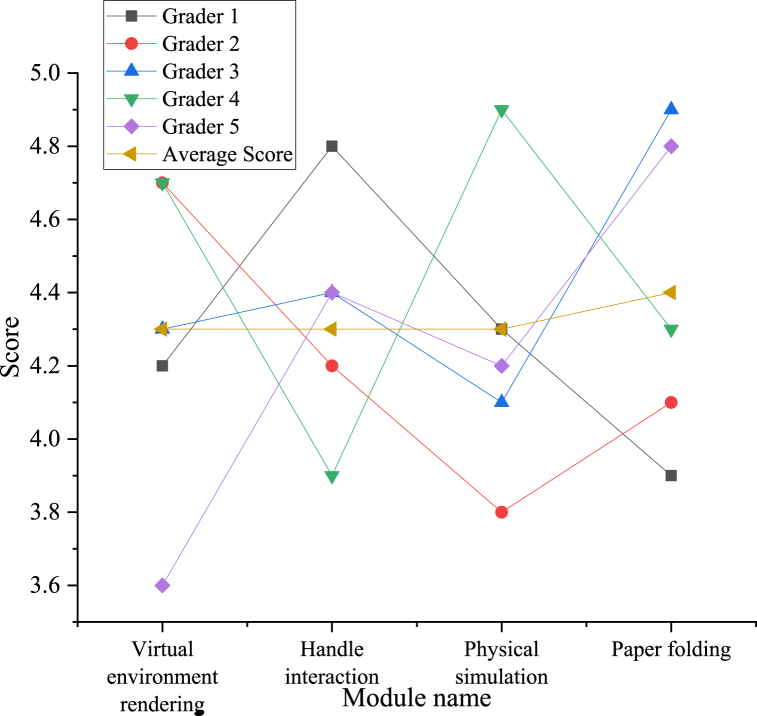
The subjective scoring results of the system.

The data results presented in Fig. 11 thoroughly validate the performance and effectiveness of each module within the proposed paper-cutting VR interactive simulation system. Specifically, the virtual environment rendering module attains an average score of 4.3 points, demonstrating its ability to produce high-quality visual effects in constructing the paper-cutting virtual environment, thus creating a realistic and captivating paper-cutting world for participants. Similarly, the user interaction module garners an average score of 4.3 points, showcasing its commendable performance in facilitating interaction between users and the virtual environment. Participants experience seamless engagement with the system through controllers, ensuring smooth operations and prompt feedback, thereby enhancing the overall user experience. The physics simulation module achieves an average score of 4.3 points, affirming the precision and realism of its simulation of physical interaction during paper-cutting processes. It accurately replicates elements such as paper texture, curvature, and cutting effects, creating an authentic experience comparable to physical paper-cutting operations. Additionally, the paper folding module merits an average score of 4.4 points, slightly surpassing other modules in performance evaluation. This underscores the remarkable precision and realism achieved by this module in simulating the paper folding process. Participants can effortlessly simulate various complex paper-folding effects using this module, thereby broadening the creative possibilities in paper-cutting creations. Furthermore, the scores assigned to these modules indicate stable and high levels of recognition and satisfaction among participants regarding their performance and effects. This unequivocally validates the proposed system's advantages and effectiveness in enriching participants' experience of paper-cutting culture. In conclusion, the research results not only validate the high performance and effectiveness of each module in the proposed system but also underscore the pivotal role of this system in enhancing participants' experience of paper-cutting culture. This system introduces novel avenues and possibilities for the inheritance and development of paper-cutting culture, thereby fostering greater understanding and appreciation of this traditional art form among a wider audience.

3) The effect of paper-cutting cultural protection under different methods

According to the questionnaire results, the detailed comparison between paper-cutting cultural preservation scores using traditional methods and VR technology is detailed in Table 10.

**Table 10:** Comparative score of protection methods.

Characteristics	VR technology protection methods	Traditional paper-cutting cultural protection methods
Immersive experience scores	4.8	3.5
Digital protection and dissemination scores	4.9	2.7
Visualization and interactivity scores	4.7	3.0
Global communication scores	4.9	3.2
Real-time feedback and improvement scores	4.6	3.1

Table 10 demonstrates that VR technology's methods in conserving paper-cutting culture have garnered high scores in immersive experience, digital preservation and dissemination, global exchange, and real-time feedback and improvement, scoring 4.8, 4.9, 4.9, and 4.6, respectively. These scores substantiate the considerable advantages of VR technology in delivering immersive experiences, fostering global cultural exchange, and facilitating real-time feedback and improvement. VR technology can immerse participants in a virtual paper-cutting environment, allowing them to deeply experience the charm of paper-cutting culture. Additionally, VR technology facilitates the digital preservation and dissemination of paper-cutting culture, transcending geographical boundaries and enabling a broader appreciation of this art form. Meanwhile, it excels in collecting real-time feedback from participants, providing robust support for enhancing and innovating paper-cutting techniques. In contrast, traditional methods of conserving paper-cutting culture receive lower scores in these aspects, scoring 3.5, 2.7, 3.2, and 3.1, respectively. This emphasizes the considerable potential for enhancing traditional methods in these domains. Conventional approaches to preserving paper-cutting culture typically rely on physical exhibitions and teaching methods, which may fall short of delivering immersive experiences. Furthermore, the dissemination of paper-cutting culture is constrained by geographical and demographic factors, impeding global exchange and dissemination efforts. Moreover, traditional methods lack effective feedback mechanisms, hindering the understanding of participants' needs and opinions and subsequent targeted improvements. In summary, the research findings highlight the substantial advantages of VR technology in preserving paper-cutting culture. VR technology addresses the limitations of traditional methods and introduces new opportunities for the heritage and advancement of paper-cutting culture. By leveraging VR technology, participants can engage in more immersive experiences, facilitate digital preservation and dissemination of paper-cutting traditions, foster global cultural exchange, and enhance paper-cutting techniques through continuous real-time feedback and refinement. The final test results of the proposed model are shown in Table 11.

**Table 11:** The final test results of the proposed model.

Test project	Result 1	Result 2	Result 3	Result 4	Result 5	Result 6
Rapid response	10 ms	8 ms	12 ms	15 ms	–	–
High throughput	120 times/min	60 times/min	–	–	–	–
Enhanced interactivity	8 ms	90fps	60 times/min	4.2 (Score for ease of operation)	4.1 (Score for personalization level)	–
The accurate simulation of physical effects by VR modules	12 ms	4.3 points	15 ms	4.4 points	90 % (Physical simulation texture similarity)	±1° (Accuracy of paper folding angle)
Comparison of experimental time	4 weeks/4.5 weeks	2 weeks/2.1 weeks	3 weeks/3.2 weeks	–	–	–
Comparison between the experimental group and the control group	4.6 points/4.2 points (cultural understanding)	4.2 points/below 4.2 points (interest)	4.5 points/below 4.5 points (system satisfaction)	4.7 points/4.3 points (content richness)	4.4 points/below 4.4 points (production process acceptance)	–
Comparison between VR and traditional methods	4.8 points/3.5 points (immersive experience)	4.9 points/2.7 points (digital protection communication)	4.7 points/3.0 points (visual interaction)	4.9 points/3.2 points (global communication)	4.6 points/3.1 points (real-time feedback improvement)	–

In Table 11, the data results presented fully demonstrate the paper-cutting VR interactive simulation system's excellent performance and remarkable advantages in many aspects, which has made significant contributions to the inheritance and development of paper-cutting culture. In terms of rapid response, the response time of each module of the system is extremely short, virtual environment rendering is 10 ms, user interaction is only 8 ms, physical simulation is 12 ms, and paper folding is 15 ms. This ensures that the system can respond quickly to user instructions, achieve real-time interaction, and greatly enhance the user experience, enabling people to feel a smooth operating environment when using the system. High throughput data is also very impressive, with virtual environment rendering throughput of 120 times/min, and user interaction throughput of 60 times/min. This means that the system can quickly handle a large number of interaction requests, meet the needs of frequent operations of users, and further enhance the system's interactivity and practicability. The enhanced interactivity is outstanding, the user interaction response time is short, the virtual environment rendering frame rate is high, and the throughput is impressive. Meanwhile, the scores for the ease of operation and personalized level are relatively high. This shows that the system is excellent in interaction design, and users can easily carry out various operations and personalize them according to their needs, which greatly increases the user's participation and satisfaction. The accurate simulation of physical effects by VR modules is remarkable, the response time of physical simulation is short, and the score is high. Additionally, the texture similarity is 90 %, and the paper folding response time and rating are also ideal, with an angle accuracy of ±1°. This allows users to truly experience the physical effects of paper-cutting through the system, providing strong technical support for the inheritance and promotion of paper-cutting culture. Although the actual time of the experiment is slightly higher than the estimated time, the overall difference is small, indicating that the experimental process is planned reasonably, and the research team has certain abilities in time management and task arrangement. In the comparison between the experimental and control groups, the average scores of the experimental group in cultural understanding, interest, system satisfaction, content richness, and production process acceptance are higher than those of the control group, which fully proves that the system has obvious advantages in promoting the spread of paper-cutting culture, can improve users' understanding and interest in this culture, and improve users' satisfaction with the system and acceptance of the production process. The comparison between VR and traditional methods shows that the scores of VR technology protection methods in immersive experience, digital protection communication, visual interaction, global communication, and real-time feedback improvement are higher than those of traditional methods. It illustrates that VR technology has great potential in the protection of paper-cutting culture, and provides new ideas and methods for protecting and developing this culture. In summary, the paper-cutting VR interactive simulation system has opened up a new way for the inheritance and development of paper-cutting culture through its excellent performance and advantages and has important practical significance and value.

## 5 Discussion

The experimental data of the paper-cutting VR interactive simulation system are comprehensively and deeply analyzed, and the system's remarkable achievements and unique contributions to improving the paper-cutting cultural experience are fully demonstrated. Firstly, the high-performance virtual environment rendering module furnishes users with a smooth and realistic visual feast, allowing them to immerse themselves fully in the world of paper-cutting. The advantages of low response time and high FPS greatly enhance the system's real-time performance and offer users a natural and comfortable visual experience. Regarding user interaction, the system performs exceptionally well. Its fast response and high throughput enable users to effortlessly perform various paper-cutting operations, such as folding and cutting, achieving a highly natural and seamless interactive experience. This enhancement in interactivity not only increases user engagement but also subtly deepens their understanding and interest in paper-cutting culture. Moreover, the precise simulation provided by the physics and origami modules is commendable. These modules accurately replicate the physical effects of paper bending, folding, and cutting during the paper-cutting process, providing users with a near-realistic paper-cutting experience. This realistic simulation not only enhances user interest and satisfaction but also offers robust technical support for the protection and promotion of paper-cutting as ICH. Across multiple evaluation dimensions, including cultural understanding, interest, system satisfaction, content richness, and acceptance of the production process, the experimental group's results significantly outperform those of the control group. These results fully demonstrate the proposed system's effectiveness in enhancing users' cultural experience of paper-cutting and have received high recognition from professional evaluations. Despite the remarkable achievements of the system, there is still room for improvement and expansion. For example, personalized interaction and feedback mechanisms are implemented to meet a wider range of user needs and preferences. Strengthening social interaction and collaborative features can further enhance user engagement and experience. Exploring the application of VR technology in education and training for ICH protection also represents a promising direction for future exploration.

Compared to the study by Eman et al. (2023), the proposed system has several advantages [45]. While their approach focuses solely on using pre-trained mask detection and segmentation technologies, this study not only employs advanced technical methods but also conducts more refined data collection and processing [46]. Furthermore, this study emphasizes expanding into practical application scenarios, better adapting to complex and changing real-world demands, and providing more practical solutions for related fields. Simultaneously, this system achieves deep interaction between users and the paper-cutting process through innovative VR technology, offering a more immersive experience [47]. While perhaps lacking in the realism of simulation or the convenience of user interaction compared to some studies, this system excels in the accuracy of physical simulation and response speed, allowing users to experience the charm of paper-cutting more realistically. Additionally, unlike studies limited to single functionalities or application scenarios, this system holds potential application value across multiple domains such as education, entertainment, and cultural heritage. In short, the proposed system stands out and makes remarkable contributions to enhancing the cultural experience of paper-cutting, injecting new vitality into the protection and inheritance of ICH [48]. With ongoing technological advancements and continual system optimization, this system is poised to play a more significant role in the future, making greater contributions to the preservation and dissemination of paper-cutting culture.

## 6 Conclusion

Comparative experiments and professional evaluations show prominent advantages of this system in enhancing users' cultural experience of paper-cutting, garnering widespread recognition and high acclaim. This innovative technological approach and solution not only offer strong support for the digital preservation of paper-cutting as ICH but also provide valuable insights and references for the application of VR technology in other cultural heritage domains. The research achievements have profound implications for the digital preservation of paper-cutting culture, showcasing the immense potential of VR technology in cultural dissemination and innovation. The system breaks through temporal and spatial limitations, enabling more people to personally experience and understand the charm of paper-cutting art, thereby significantly promoting its widespread dissemination. However, this study also has certain limitations. Firstly, the relatively small sample size may limit the research results' universality and applicability to some extent. In the future, it is planned to expand the sample size and increase the diversity of the sample to improve the study's reliability. Secondly, the short duration of experiments may not fully reflect the long-term effects of system usage. Subsequent research could consider extending the experimental period to evaluate the system's sustained performance and long-term user engagement. Looking ahead, the application of VR technology in the ICH protection field continues to expand, particularly in traditional music, dance, and drama, injecting new vitality into more cultural treasures.

## Data availability statement

Data will be made available on request.

## CRediT authorship contribution statement

**Lulu Zhao:** Writing – original draft, Resources, Methodology, Investigation, Formal analysis, Data curation, Conceptualization. **JaeWoong Kim:** Writing – review & editing, Visualization, Validation, Supervision, Software, Resources, Project administration.

## Declaration of competing interest

The authors declare that they have no known competing financial interests or personal relationships that could have appeared to influence the work reported in this paper.
