# Supplementary material for: The impact of traditional Chinese paper-cutting in digital protection for intangible cultural heritage under virtual reality technology
Source: Heliyon. 2024 Sep 18;10(18):e38073. doi: 10.1016/j.heliyon.2024.e38073 (PMC11425177; doi:10.1016/j.heliyon.2024.e38073)
Supplement: Multimedia component 1 [file mmc1.docx]

**Survey Questionnaire**

Dear Participant,

Thank you for participating in our paper-cutting cultural experience experiment! Your feedback is crucial for improving our paper-cutting VR interactive simulation system and understanding the paper-cutting cultural experience better. Please share your opinions on system performance, understanding of paper-cutting culture, experience process, and system modules in the following questionnaire. Your honest responses will help us optimize the system to provide a more enhanced cultural experience. Please fill out the questionnaire truthfully; your personal information will only be used for research purposes, and we will ensure your privacy. Thank you for your participation and support!

1) Your age (Single choice)

A. 18-24 years old

B. 25-34 years old

C. 35-44 years old

D. 45-54 years old

E. 55 years old and above

2) Your gender (Single choice)

A. Male

B. Female

3) Do you have any experience with paper-cutting? (Single choice)

A. Yes

B. No

4) How much do you know about paper-cutting culture? (Rate from 1 to 5)

A. 1 - No understanding

B. 2 - Poor understanding

C. 3 – General understanding

D. 4 - Good understanding

E. 5 - Very good understanding

5) How interested are you in the VR paper-cutting cultural experience project? (Rate from 1 to 5)

A. 1 - Not interested at all

B. 2 - Not very interested

C. 3 - Moderately interested

D. 4 - Interested

E. 5 - Very interested

6) How satisfied are you with the virtual environment rendering in the VR paper-cutting cultural experience project? (Rate from 1 to 5)

A. 1 - Very dissatisfied

B. 2 - Dissatisfied

C. 3 - Moderately satisfied

D. 4 - Satisfied

E. 5 - Very satisfied

7) How satisfied are you with the controller interaction in the VR paper-cutting cultural experience project? (Rate from 1 to 5)

A. 1 - Very dissatisfied

B. 2 - Dissatisfied

C. 3 - Moderately satisfied

D. 4 - Satisfied

E. 5 - Very satisfied

8) How satisfied are you with the physical simulation in the VR paper-cutting cultural experience project? (Rate from 1 to 5)

A. 1 - Very dissatisfied

B. 2 - Dissatisfied

C. 3 - Moderately satisfied

D. 4 - Satisfied

E. 5 - Very satisfied

9) How satisfied are you with the paper folding module in the VR paper-cutting cultural experience project? (Rate from 1 to 5)

A. 1 - Very dissatisfied

B. 2 - Dissatisfied

C. 3 - Moderately satisfied

D. 4 - Satisfied

E. 5 - Very satisfied

10) How rich do you think the paper-cutting content presented by the paper-cutting VR interactive simulation system is? (Rate from 1 to 5)

A. 1 - Not very rich

B. 2 - Somewhat rich

C. 3 - Moderately rich

D. 4 - Quite rich

E. 5 - Very rich

11) Please list the part of the content presented by the system that attracted you the most. (fill in the blank)

12) If you think the content presented by the system is not rich enough, what aspects could be improved? (fill in the blank)

13) When experiencing the paper-cutting production process in the VR interactive simulation system, how satisfied are you with this process? (Rate from 1 to 5)

A. 1 - Very dissatisfied

B. 2 - Dissatisfied

C. 3 - Moderately satisfied

D. 4 - Satisfied

E. 5 - Very satisfied

14) How helpful do you think the paper-cutting VR interactive simulation system is during the paper-cutting production process? (Rate from 1 to 5)

A. 1 - Not helpful at all

B. 2 - Not very helpful

C. 3 - Moderately helpful

D. 4 - Quite helpful

E. 5 - Very helpful

15) Please describe your favorite feature or experience during the paper-cutting production process. (fill in the blank)

16) If you think there is room for improvement in the VR paper-cutting interactive simulation system during the paper-cutting production process, what suggestions would you make? (fill in the blank)

17) Briefly describe the level of realism you felt in the virtual environment. (fill in the blank)

18) Briefly describe which aspects of the system presentation you are satisfied with. (fill in the blank)

19) Briefly describe your feelings when experiencing the VR paper-cutting interactive simulation system (or traditional paper-cutting teaching). (fill in the blank)

20) Which teaching method do you think is more helpful for your learning?

A. Paper-cutting VR interactive simulation system

B. Traditional paper-cutting teaching

C. Both are helpful

D. Neither is helpful

E. Not sure

Thank you for taking the time to fill out this questionnaire. Your opinions are crucial for improving the paper-cutting VR interactive simulation system and enhancing the cultural experience of paper-cutting. Your participation allows us to gain a deeper understanding of the system's strengths and areas for improvement, providing valuable guidance for future research and development. If you have any additional opinions, suggestions, or experiences you would like to share, please feel free to contact us. We will carefully consider each piece of feedback to ensure our system better meets your expectations. Thanks again for your support and participation! We look forward to continuing to collaborate with you in future research. Best wishes for your success!
